# *NEAT1* Confers Radioresistance to Hepatocellular Carcinoma Cells by Inducing Autophagy through GABARAP

**DOI:** 10.3390/ijms23020711

**Published:** 2022-01-10

**Authors:** Hiromi Sakaguchi, Hiroyuki Tsuchiya, Yutaka Kitagawa, Tomohiko Tanino, Kenji Yoshida, Nobue Uchida, Goshi Shiota

**Affiliations:** 1 Department of Radiation Oncology, Tottori University Hospital, 86 Nishi-cho, Yonago 683-8503, Japan; sakaguchi0130@tottori-u.ac.jp (H.S.); ykitagawa@tottori-u.ac.jp (Y.K.); t_tanino217@yahoo.co.jp (T.T.); kyoshi@tottori-u.ac.jp (K.Y.); 2 Division of Medical Genetics and Regenerative Medicine, Department of Genomic Medicine and Regenerative Therapeutics, Graduate School of Medicine, Tottori University, 86 Nishi-cho, Yonago 683-8503, Japan; gshiota@tottori-u.ac.jp; 3 Radiation Therapy, Tokyo Saiseikai Central Hospital, 1-4-17 Mita, Minato-ku, Tokyo 108-0073, Japan; nobueuchida@gmail.com

**Keywords:** *NEAT1*, GABARAP, autophagy, radioresistance, hepatocellular carcinoma, long noncoding RNA

## Abstract

A long noncoding RNA (lncRNA), *nuclear enriched abundant transcript 1* (*NEAT1*) *variant 1* (*NEAT1v1*), is involved in the maintenance of cancer stem cells (CSCs) in hepatocellular carcinoma (HCC). CSCs are suggested to play important roles in therapeutic resistance. Therefore, we investigated whether *NEAT1v1* is involved in the sensitivity to radiation therapy in HCC. Gene knockdown was performed using short hairpin RNAs, and *NEAT1v1*-overexpressing HCC cell lines were generated by stable transfection with a *NEAT1v1*-expressing plasmid DNA. Cells were irradiated using an X-ray generator. We found that *NEAT1* knockdown enhanced the radiosensitivity of HCC cell lines and concomitantly inhibited autophagy. *NEAT1v1* overexpression enhanced autophagy in the irradiated cells and conferred radioresistance. Gamma-aminobutyric acid receptor-associated protein (GABARAP) expression was downregulated by *NEAT1* knockdown, whereas it was upregulated in *NEAT1v1*-overexpressing cells. Moreover, GABARAP was required for *NEAT1v1*-induced autophagy and radioresistance as its knockdown significantly inhibited autophagy and sensitized the cells to radiation. Since GABARAP is a crucial protein for the autophagosome-lysosome fusion, our results suggest that *NEAT1v1* confers radioresistance to HCC by promoting autophagy through GABARAP.

## 1. Introduction

Primary liver cancer is a malignant tumor with a poor prognosis and the third leading cause of cancer death worldwide [1]. In particular, hepatocellular carcinoma (HCC) accounts for 80% of primary liver cancers and is the most common cause of liver cancer death [1]. Curative treatments for early-stage HCC are surgical resection, liver transplantation, and radiofrequency ablation [2]. However, most HCC patients are diagnosed at intermediate and advanced stages and do not benefit from these treatments [3]. Therefore, patients with locally advanced HCC are mainly treated with transcatheter arterial chemoembolization. Recently, stereotactic body radiation therapy has emerged as another local ablative noninvasive treatment approach with high rates of local control [4]. However, the emergence of radioresistant tumor cells is still a major obstacle for the clinical application of radiotherapy as a treatment against HCC. Therefore, it is essential to improve our understanding of the mechanisms underlying HCC radioresistance to increase the efficacy of radiotherapy.

Cancer stem cells (CSCs) constitute a subpopulation of cancer cells capable of self-renewal and differentiation into non-CSCs [5,6]. Moreover, CSCs are likely resistant to chemotherapy and radiation therapy and might consequently be involved in tumor recurrence and metastasis [5,7,8]. In HCC, CD44, CD133, and epithelial adhesion molecules have been identified as CSC markers. The expression levels of these markers were associated with poor prognosis [5].

*Nuclear enriched abundant transcript 1* (*NEAT1*) is a long noncoding RNA (lncRNA) required for the formation of paraspeckles, which are nuclear substructures found in most cultured cells [9]. The *NEAT1* gene is expressed as two variant isoforms: *NEAT1v1* (3.8 kb in length in humans) and *NEAT1v2* (22.7 kb). We previously reported that *NEAT1v1* plays a critical role in the maintenance of CSC properties in HCC [10]. We also demonstrated that *NEAT1v1*-overexpressing HCC cells were resistant to 5-fluorouracil and cisplatin, whereas the knockout (KO) of *NEAT1* increased the cell sensitivity to those drugs [10]. However, the association between radiosensitivity of HCC and *NEAT1v1* remains to be determined.

In the present study, we investigated the effect of *NEAT1v1* on HCC cell radiosensitivity. Our data revealed that *NEAT1v1* induced radioresistance in HCC cells. We also demonstrated that the radioresistance was mediated by autophagy via gamma-aminobutyric acid A receptor type A-associated protein (GABARAP) and that *NEAT1v1* induced the expression of GABARAP at the transcription level in HCC cells. These results suggest that *NEAT1v1* contributes to the establishment of HCC resistance to radiotherapy.

## 2. Results

### 2.1. Radiosensitization of HCC Cell Lines by NEAT1 Knockdown

**Figure 1:**
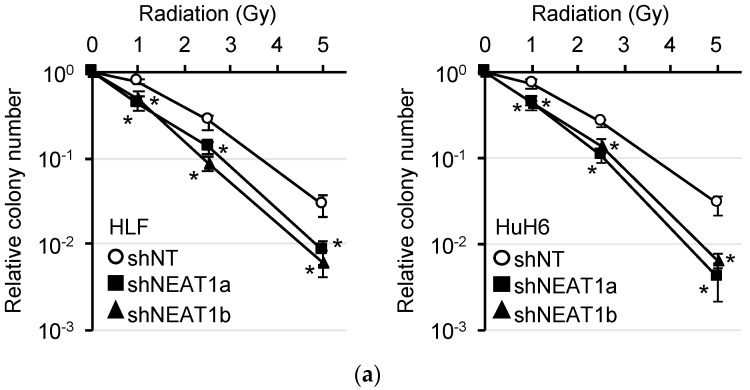

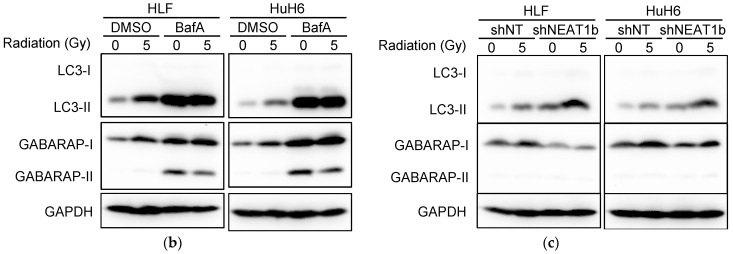
Radiosensitization induced by *NEAT1* knockdown in hepatocellular carcinoma (HCC) cell lines. (**a**) Clonogenicity of HLF (left) and HuH6 (right) after X-ray irradiation. Following irradiation, cells were transduced with adenovirus vectors expressing non-targeting (shNT) (open circles) or *NEAT1*-targeting shRNAs (shNEAT1a and shNEAT1b; closed rectangles and triangles, respectively). * *p* < 0.05 vs. shNT; Dunnett’s test (*n* = 4). (**b**,**c**) LC3 protein expression in HLF and HuH6 cells irradiated at 5 Gy. (**b**) Twenty-four hours after irradiation, cells were treated with dimethyl sulfoxide (DMSO) or 50 nM bafilomycin A1 (BafA) for 24 h. (**c**) Following irradiation, cells were immediately transduced with shNT or shNEAT1b adenovirus vectors for 48 h. GAPDH was used as an internal control.

*NEAT1v1* and *NEAT1v2* have the same transcription start site, but their transcription terminates at different positions without splicing (Figure S1A). The expression of total *NEAT1* (*NEAT1v1* and *NEAT1v2*) and *NEAT1v2* was significantly knocked down by shRNAs targeting NEAT1 (shNEAT1a and shNEAT1b) in HLF and HuH6 cell lines (Figure S1B). To evaluate the radiosensitivity of these cells, we performed a colony formation assay. It showed that the number of colonies was significantly decreased by *NEAT1* knockdown (Figure 1a).

### 2.2. Stronger Suppression of Autophagy Induced by Radiation after NEAT1 Knockdown

Autophagy is one of the factors determining radiosensitivity [11]. LC3-II levels were increased in HLF and HuH6 cells after 5-Gy irradiation. However, they were not affected by the presence of bafilomycin A1 (BafA) (Figure 1b and Figure S2A), suggesting that the autophagy flux was inhibited by radiations. GABARAP, similar to LC3-II, is incorporated into autophagosomes and degraded by autophagy. GABARAP expression was also upregulated by radiation (Figure 1b and Figure S2A). *NEAT1* knockdown further increased LC3-II levels in irradiated cells, suggesting that it enhanced the suppression of autophagy induced by radiations (Figure 1c and Figure S2B). The expression of GABARAP was decreased by *NEAT1* knockdown in irradiated cells (Figure 1c and Figure S2B).

### 2.3. Suppression of Autophagy by NEAT1 Knockout

**Figure 2:**
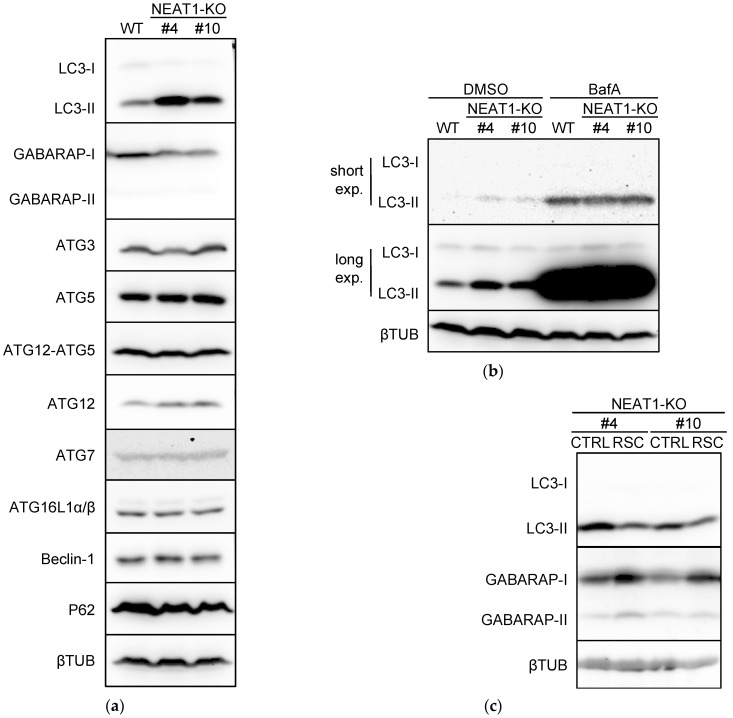
Impaired autophagy in *NEAT1* knockout (*NEAT1*-KO) HuH7 cells. (**a**) Expression of autophagy-related proteins in parental HuH7 wild-type (WT) or *NEAT1*-KO (#4 and #10) cells. (**b**) LC3 protein expression (upper; short exposure; lower; long exposure) in *NEAT1*-KO cells treated with dimethyl sulfoxide (DMSO) or 50 nM bafilomycin A1 (BafA) for 24 h. (**c**) LC3 and GABARAP expression in *NEAT1*-KO (CTRL) and rescued (RSC) cells. The internal control was βTUB.

Because *NEAT1* knockdown affected the autophagy flux (Figure 1), we investigated the expression of autophagy-related proteins in *NEAT1*-KO cells (clones #4 and #10) [10]. The expression of LC3-II and autophagy-related gene (ATG) 12 was upregulated in *NEAT1*-KO cells, whereas that of GABARAP was downregulated (Figure 2a and Figure S2C). Although ATG12 exerts its function by conjugating with ATG5 [12], the expression of the ATG12-ATG5 complex was not affected by *NEAT1*-KO (Figure 2a and Figure S2C). The autophagic flux assay revealed that the difference in LC3-II expression between wild-type (WT) and *NEAT1*-KO cells disappeared in the presence of BafA (Figure 2b and Figure S2D), suggesting that autophagy was suppressed in *NEAT1*-KO cells. This notion is consistent with the fact that GABARAP is a protein essential for the formation of autolysosomes and that its downregulation suppresses autophagy [13,14]. Next, we examined the expression levels of LC3-II and GABARAP in RSC cells generated from *NEAT1*-KO cells by stable transfection with *NEAT1v1*-expressing plasmid DNA. As shown in Figure 2c and Figure S2E, LC3-II levels were decreased by *NEAT1v1* overexpression in *NEAT1*-KO cells, whereas GABARAP expression was increased. These results suggest that GABARAP protein expression is regulated by *NEAT1v1*.

**Figure 3:**
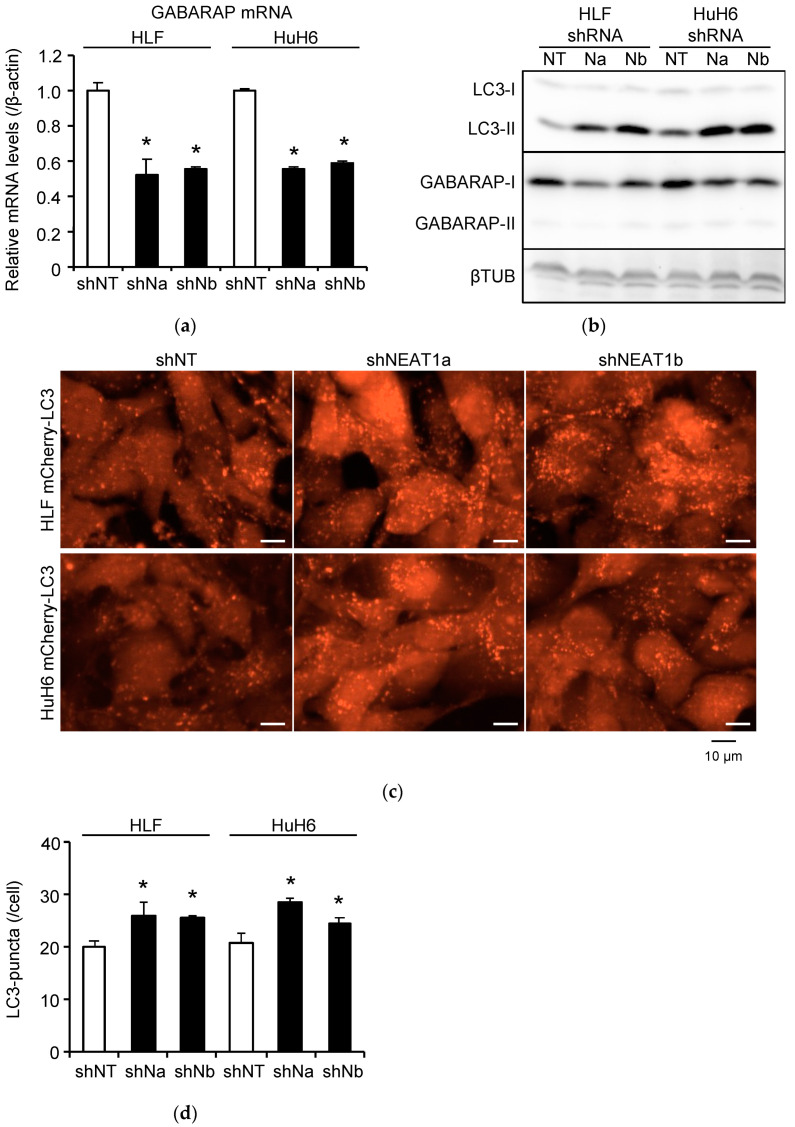
Inhibition of autophagy by *NEAT1* knockdown in HCC cell lines. (**a**) *GABARAP* mRNA expression levels in HLF and HuH6 cells transduced with shNT, shNEAT1a (shNa), or shNEAT1b (shNb) for 48 h. * *p* < 0.05 vs. shNT; Dunnett’s test (*n* = 3). (**b**) LC3 and GABARAP protein expression in HLF and HuH6 cells transduced with shNT (NT), shNEAT1a (Na), or shNEAT1b (Nb) for 48 h. The internal control was βTUB. (**c**,**d**) Representative images (**c**) and relative number (**d**) of LC3 puncta in mCherry-LC3-expressing HLF and HuH6 cells transduced with shNT, shNEAT1a (shNa), or shNEAT1b (shNb) for 48 h. * *p* < 0.05 vs. shNT; Dunnett’s test (*n* = 4).

As NEAT1 is required for the maintenance of CSCs in HCC cell lines [10], the constitutive lack of this lncRNA might cause unexpected phenotypic changes. Thus, we investigated the effects of *NEAT1* deficiency on autophagy by performing transient knockdown of *NEAT1*. As shown in Figure 3a,b and Figure S2F, *NEAT1* knockdown decreased the expression levels of GABARAP in HLF and HuH6 cells at both mRNA and protein levels. A reporter assay measuring the activity of the GABARAP promoter indicated that *NEAT1v1* induced GABARAP expression by activating its promoter (Figure S3), while the intracellular stability of GABARAP protein was not affected by *NEAT1v1* (Figure S4), suggesting that *NEAT1v1* regulates GABARAP expression at the transcriptional level. Moreover, *NEAT1* knockdown significantly increased LC3-II expression level and the number of LC3 puncta (Figure 3b–d and Figure S2F). These results suggest that *NEAT1* is involved in maintaining the autophagy flux in HCC cells under nonirradiated conditions.

### 2.4. Induction of Autophagy and Radioresistance by NEAT1v1

**Figure 4:**
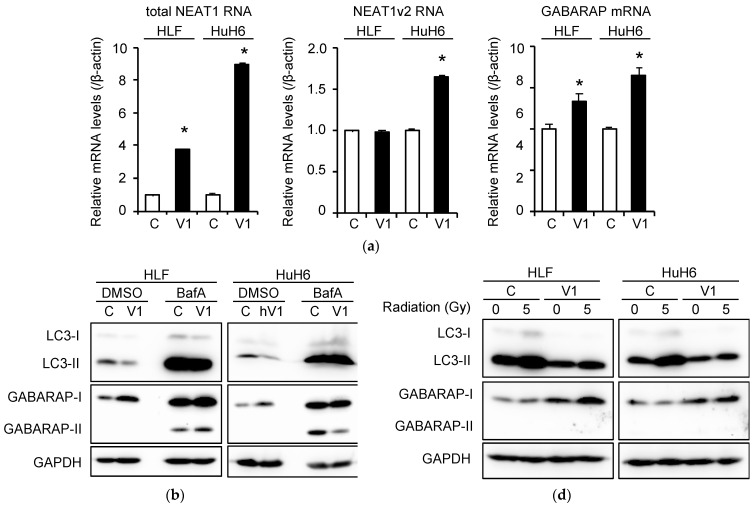

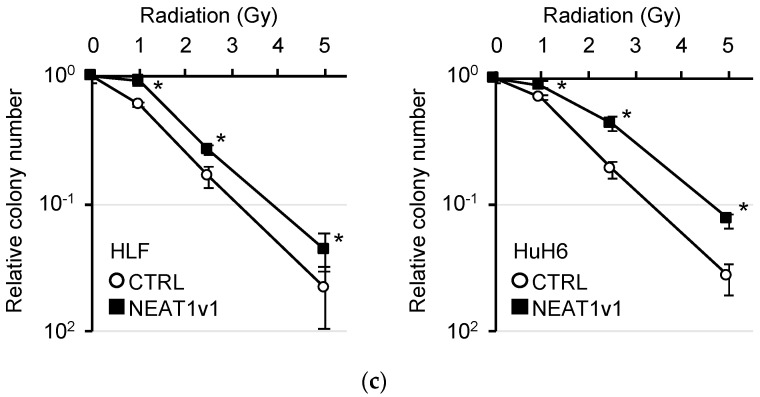
Promotion of autophagy and radioresistance by *NEAT1v1* overexpression. (**a**) Expression levels of total *NEAT1*, *NEAT1v2*, and *GABARAP* mRNA in HLF and HuH6 cells stably transfected with *NEAT1v1* (V1) or empty vectors (C). * *p* < 0.05 vs. C; Students’ *t*-test (*n* = 4). (**b**) LC3 and GABARAP protein expression in *NEAT1v1*-expressing HLF and HuH6 cells treated with DMSO or 50 nM bafilomycin A1(BafA) for 24 h. (**c**) Clonogenicity of HLF (left) and HuH6 (right) cells after X-ray irradiation. Cells were stably transfected with *NEAT1v1* (closed rectangles) or empty (CTRL; open circles) vectors. * *p* < 0.05 vs. CTRL; Students’ *t*-test (*n* = 3). (**d**) LC3 and GABARAP protein expression in *NEAT1v1*-expressing HLF and HuH6 cells 48 h after 5-Gy irradiation.

We previously reported that *NEAT1v1*, but not *NEAT1v2*, is required for the maintenance of CSCs in HCC cell lines [10]. The rescue experiment showed that the overexpression of *NEAT1v1* increased GABARAP levels in *NEAT1*-KO cells (Figure 2c and Figure S2E). Thus, HLF and HuH6 cell lines stably expressing *NEAT1v1* were generated. In these cells, the mRNA expression levels of total *NEAT1* and GABARAP were significantly increased (Figure 4a). Although no significant difference in *NEAT1v2* expression was observed in HLF cells, it was approximately 1.6-fold upregulated by *NEAT1v1* overexpression in HuH6 cells (Figure 4a). However, in both cell lines, GABARAP protein expression was upregulated, whereas that of LC3-II was downregulated, suggesting that autophagy was promoted by *NEAT1v1* overexpression (Figure 4b and Figure S2G). Radioresistance was also significantly increased by *NEAT1v1* overexpression in both cell lines (Figure 4c). Moreover, as shown in Figure 4d and Figure S2H, LC3-II protein expression was downregulated even after irradiation, suggesting that the autophagy flux inhibited by irradiation was restored by *NEAT1v1* overexpression.

### 2.5. Induction of CSC Marker Expression by NEAT1v1 and Radiation

Because *NEAT1v1* is involved in the maintenance of CSCs and was shown to regulate CD44, a CSC marker, expression in HuH7 and HepG2 cell lines [10], we determined the expression of CSC markers in HLF and HuH6 cell lines stably expressing *NEAT1v1* after irradiation. As shown in Figure S5, the expression of total *NEAT1*, *NEAT1v2,* and *GABARAP* was not changed by the radiation. *CD13* mRNA was upregulated after irradiation in both cell lines overexpressing *NEAT1v1* (Figure S5). However, the expression of *CD44* was increased only in *NEAT1v1*-overexpressing HLF cells, while radiation upregulated its expression in both cell lines (Figure S5). By contrast, *CD90* was downregulated in *NEAT1v1*-overexpressing HLF cells, while it was significantly upregulated in *NEAT1v1*-overexpressing HuH6 cells after irradiation (Figure S5). The expression of the *epithelial cell adhesion molecule* (*EPCAM*) was induced by radiation in HLF cells but not affected by *NEAT1v1* (Figure S5). Of note, *CD133* expression could not be detected because of its low expression in the cell lines (data not shown). These results suggest that *NEAT1v1* induces CSCs expressing different CSC markers depending on the HCC cell lines.

### 2.6. Induction of Radioresistance by NEAT1 via GABARAP

**Figure 5:**
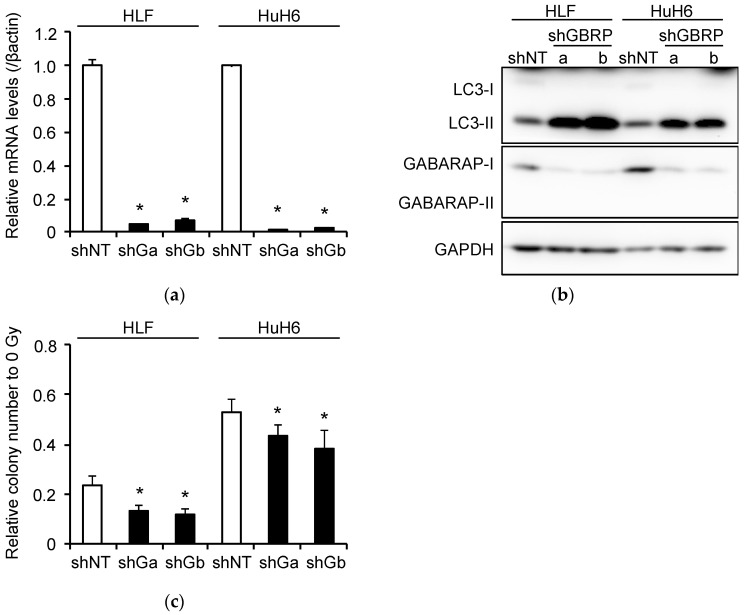
Radiosensitization and autophagy inhibition induced by *GABARAP* knockdown in HCC cell lines. (**a**) *GABARAP* mRNA expression levels in HLF and HuH6 cells transduced with shNT or *GABARAP*-targeting shRNAs (shGa and shGb) for 48 h. * *p* < 0.05 vs. shNT; Dunnett’s test (*n* = 3). (**b**) LC3 and GABARAP protein expression in HLF and HuH6 cells transduced with shNT or *GABARAP*-targeting shRNAs (shGBRPa and shGBRPb) for 48 h. (**c**) Clonogenicity of HLF and HuH6 overexpressing *NEAT1v1* after X-ray irradiation. Following irradiation, the cells were immediately transduced with shNT, shGa, or shGb. * *p* < 0.05 vs. shNT; Dunnett’s test (*n* = 4).

GABARAP expression was significantly knocked down by shRNAs targeting GABARAP (shGBRPa and shGBRPb) in HLF and HuH6 cells overexpressing *NEAT1v1* (Figure 5a,b and Figure S2I). Concomitantly, LC3-II protein levels were markedly increased, suggesting the suppression of autophagy flux (Figure 5b and Figure S2I). Moreover, the clonogenicity of *NEAT1v1*-overexpressing HLF and HuH6 cells after irradiation was significantly decreased by GABARAP knockdown (Figure 5c). These results suggest that GABARAP is a critical mediator for *NEAT1v1*-induced radioresistance.

### 2.7. Relationship between GABARAP Expression in Tissues from HCC Patients and Prognosis

**Figure 6:**
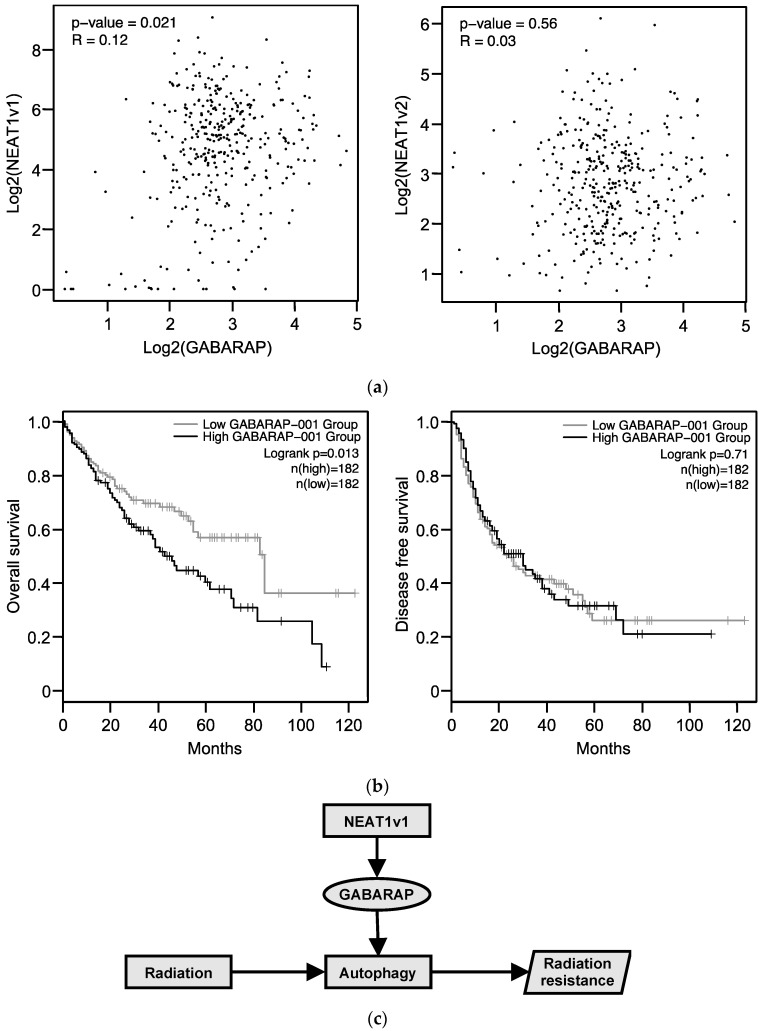
*NEAT1* and *GABARAP* expression in HCC tissues of the TCGA dataset. (**a**) Spearman correlation analysis between *GABARAP* (ENST00000302386.9) and *NEAT1v1* (ENST00000499732.2) (left) or *NEAT1v2* (ENST00000501122.2) (right). (**b**) Overall and recurrence-free survival of HCC patients according to *GABARAP* (ENST00000302386.9) expression. (**c**) Schematic representation of the mechanism underlying *NEAT1v1*-induced radioresistance via GABARAP.

We analyzed RNA-seq data from HCC tissues obtained from TCGA. The expression of *NEAT1v1*, but not that of *NEATv2*, was significantly correlated with *GABARAP* expression (Figure 6a). Moreover, *GABARAP* expression was significantly correlated with the overall survival but not with the disease-free survival (Figure 6b). The patients registered in TCGA appear to have received various treatments, although the exact treatments are not clearly listed. However, these data suggest that autophagic dysfunctions are present in HCC tissues and might be associated with clinical outcomes in patients with HCC.

## 3. Discussion

Although radiation therapy is effective and noninvasive, it has so far played a limited role in HCC treatment because of the low tolerance of normal hepatic tissue. Recent advancements in radiation therapy, such as SBRT, allow efficiently delivering an ablative dose of radiation to tumors while sparing normal hepatocytes. Consequently, the number of HCC patients treated with radiation therapy is increasing [15]. However, radioresistance still remains a challenging issue, and its underlying mechanism needs to be clarified to improve the clinical efficacy of radiation therapy. The present study reveals that *NEAT1v1* induces radioresistance in HCC cells. Moreover, we demonstrated that GABARAP expression is induced by *NEAT1v1* and is involved in radioresistance by promoting autophagy (Figure 6c).

GABARAP, similar to LC3, belongs to the ATG8 family and is conjugated with phosphatidylethanolamine to bind the autophagosome membrane [16,17]. The ATG8 family consists of the LC3 subfamily, which includes LC3A, LC3B, and LC3C, and the GABARAP subfamily, constituted by GABARAP, GABARAPL1, and GABARAPL2 [16,17]. ATG8 family members are involved in the autophagosome formation and the autophagosome-lysosome fusion by binding proteins harboring an LC3-interacting region, including autophagy core proteins, cargo receptors, transport proteins, and proteins from the fusion machinery [16]. LC3 subfamily members are involved in the incorporation of autophagy cargos, whereas the GABARAP subfamily members are required for the autophagosome–lysosome fusion [17,18]. Our data demonstrate that the knockdown of *GABARAP* led to the inhibition of autophagy assessed by the accumulation of LC3-II (Figure 5b and Figure S2I). Therefore, and in agreement with previous studies [18], GABARAP is an indispensable protein for the formation of autolysosome.

Autophagy is an essential cellular process involving multiple factors in addition to GABARAP and LC-3. Beclin-1 is a component of class III phosphatidylinositol 3-kinase complex I and initiates autophagosome formation followed by phagophore expansion [19]. ATG3, ATG5, ATG7, ATG12, and ATG16L1 are involved in the sequential process of the conjugation of ATG8 family proteins with phosphatidylethanolamine [20,21]. P62, a representative autophagy substrate, plays a role as a cargo receptor that incorporates proteins and organelles into autophagosomes by binding to LC-3II [17]. The function of beclin-1 is inhibited by B-cell CLL/lymphoma 2, and thus, it has been demonstrated that beclin-1 acts as a tumor suppressor [22,23]. ATG proteins, such as ATG3, ATG12, and ATG16L1, are also demonstrated to have anti-oncogenic properties [24,25,26,27]. The impairment of autophagy causes the accumulation of P62, leading to the development of HCC [28]. Moreover, mice lacking *Atg5* or *Atg7* have been reported to develop liver cancers [29]. This suggests that autophagy plays a pivotal role in suppressing hepatocarcinogenesis. Moreover, GABARAP and LC3 were reported to have an anti-oncogenic function [16]. However, because autophagy plays a variety of pathophysiological roles, it contributes to both the survival and death of cancer cells [30,31]. Indeed, the expression of GABARAP and LC3 in breast cancer is significantly correlated with tumor malignancy and poor prognosis [32]. Moreover, the suppression of FOXO3 expression confers sorafenib resistance to HCC cells by accelerating autophagy [33], whereas the inhibition of autophagy enhanced the sensitivity to sorafenib [34]. Accumulating evidence suggests that autophagy has inhibitory functions against early carcinogenic processes in normal cells, whereas, in cancer cells, it contributes to tumor growth, malignant transformation, and therapeutic resistance [21]. This notion is consistent with our analysis of the TCGA dataset showing that the expression of *GABARAP* was correlated to the shortening of the overall survival of patients with HCC (Figure 5b and Figure S2I). Therefore, GABARAP-induced autophagy might promote the malignant transformation of HCC.

Autophagy is associated with the radiosensitivity of cancer cells. In esophageal cancer, autophagy induced by liver kinase B1 through the AMP-activated protein kinase pathway was shown to enhance radioresistance [35]. Additionally, cancer cells knocked down for *ATG5* or *beclin-1* exhibited an enhanced radiosensitivity as a consequence of the inhibition of autophagy [36]. In agreement with these reports, the present study demonstrated that autophagy in HCC cells was suppressed after irradiation (Figure 1b and Figure S2A). In contrast, *NEAT1v1* induced the expression of GABARAP and promoted autophagy (Figure 4d and Figure S2H). Moreover, we showed that *GABARAP* knockdown inhibited autophagy in HCC cells overexpressing *NEAT1v1* and enhanced their radiosensitivity (Figure 5b,c and Figure S2I). These results suggest that autophagy-mediated degradation and regeneration of organelles injured by radiation constitute possible mechanisms underlying the radioresistance of HCC. Our findings suggest that *NEAT1* and *GABARAP* play important roles in organelle regeneration in HCC cells and that the inhibition of *NEAT1v1* and *GABARAP* would improve the efficacy of radiation therapy. However, it was shown that autophagy accelerates radiation-induced cell death and enhances radiosensitivity [37,38,39]. The inhibition of autophagy also reduces radioresponse in vivo by suppressing the anti-tumor immunity [36]. In addition, autophagy is required to maintain homeostasis in cancer cells and in normal cells. Therefore, further studies are required to investigate the clinical benefit of radiosensitization provided by the inhibition of *NEAT1v1* and GABARAP.

Several studies showed that *NEAT1* is involved in the regulation of autophagy. *NEAT1* accelerates lipopolysaccharide-induced autophagy to enhance the inflammatory response in renal fibroblast cells and osteoblast cells [40,41]. Conversely, *NEAT1* inhibits PTEN-induced kinase 1dependent autophagy by facilitating the proteasomal degradation of NEDD4-like E3 ubiquitin-protein ligase. Consequently, *NEAT1* inhibits the degradation and regeneration of impaired mitochondria and promotes pathophysiological processes involved in Alzheimer’s disease [42]. There are controversial reports regarding the regulation of autophagy by *NEAT1* [43,44] in cardiac myocytes. The discrepancies might be attributed to the different pathologies and experimental models and require further investigation. In contrast, *NEAT1* was reported to induce autophagy to enhance drug resistance of colon cancer and HCC [45,46]. Here, we also found that *NEAT1v1* induced autophagy via GABARAP. Moreover, we demonstrated that *NEAT1v1* activated the promoter of the *GABARAP* gene (Figure S3). The precise mechanisms by which *NEAT1v1* induces *GABARAP* expression remain to be elucidated. The identification of the binding partners of *NEAT1v1* by RNA pull-down assay would facilitate accomplishing it. Other additional *NEAT1v1* target genes in HCC would also be useful to identify the transcription factors that regulate *NEAT1v1* target genes. Conversely, in the above-mentioned studies [40,43,45,46], *NEAT1* promoted autophagy by acting as a competing endogenous RNA (ceRNA) and preventing miRNAs (*miR-22-3p*, *miR-34a*, *miR-204*, and *miR-378a-3p*) to target autophagy-related proteins. Therefore, *NEAT1* might regulate GABARAP expression at the pre-and post-transcriptional levels.

*NEAT1* might influence the radiosensitivity of various cancer cells including HCC. In many cases, *NEAT1*, as a ceRNA, inhibited *miR-27b-3p* [47], *miR-101-3p* [48,49], *miR-193-3p* [50], and *miR-204* [51] to augment [47,48,50,51] or attenuate [49] the radioresistance of cancer cells. However, these miRNAs are involved in the cell cycle and epithelial-mesenchymal transition. Autophagy-related miRNAs have not been implicated in the radiosensitivity regulated by *NEAT1*. On the other hand, although the involvement of miRNAs is not clear, *NEAT1* induces radioresistance in triple-negative breast cancer (TNBC) cells by upregulating the translation of NAD(P)H:quinone oxidoreductase rather than its transcription [52]. Intriguingly, *NEAT1* concomitantly increased the cancer stemness of TNBC cells and upregulated the expression of BMI1, OCT4, and SOX2 [52]. In agreement with this, we demonstrated that *NEAT1v1* increased CSC makers, such as CD13, CD44 and CD90, in irradiated cells, suggesting that *NEAT1v1* might induce cancer stemness to protect HCC cells from radiation. Because CSCs are more dependent on autophagy than normal stem cells [53], *NEAT1v1*-induced autophagy via GABARAP might be a possible mechanism underlying the maintenance of cancer stemness.

In conclusion, we show that *NEAT1v1* confers radioresistance to HCC cells by inducing autophagy through GABARAP (Figure 6c). Thus, treatments targeting *NEAT1v1* and GABARAP are expected to improve the efficacy of radiation therapy. Moreover, since *NEAT1v1* plays a pivotal role in maintaining cancer stemness, the suppression of *NEAT1v1* might lead to effective eradication of HCC.

## 4. Materials and Methods

### 4.1. Cell Culture

Human HCC cell lines, HuH7, HLF, and HuH6, were purchased from the Japanese Collection of Research Bioresources Cell Bank (Osaka, Japan) and were maintained in Dulbecco’s Modified Eagle Medium (Nissui Pharmaceutical, Tokyo, Japan) supplemented with 10% inactivated fetal bovine serum (Sigma-Aldrich, St. Louis, MO, USA). *NEAT1*-KO HuH7 cells and rescue (RSC) cells were previously reported [10]. HLF and HuH6 cells overexpressing human *NEAT1v1* were constructed by transfection of pcDNA6-hNEAT1v1-AcGFP [10] with LipofectAMINE2000 (Thermo Fisher Scientific, Waltham, MA, USA). Following blasticidin selection, AcGFP-positive cells were sorted by flow cytometry. HLF and HuH6 cells overexpressing mCherry-LC3 were constructed by transfection of pmCherry-LC3 [54], which was provided by the RIKEN BRC through the National Bio-Resource Project of the MEXT, Japan, with LipofectAMINE2000. Following G418 selection, an mCherry-positive clone was manually picked.

### 4.2. Adenovirus Construction

All oligo DNAs and primers used are shown in Table S1. A BsaI-linker was ligated into pENTR/U6 (Thermo Fisher Scientific), resulting in pENTR/U6-Bgl2. The pAmCyan1-C1 plasmid (Takara Bio, Shiga, Japan) was digested with *Bgl*II and *Bam*HI and then self-ligated to remove the multi-cloning site, resulting in pAmCyan1-noMCS. The AmCyan1-expressing cassette was amplified by KOD-neo-plus (Toyobo, Osaka, Japan) with AmCyan primers. Following *Bam*HI digestion, it was inserted into the *Bgl*II site of pENTR/U6-Bgl2, resulting in pENTR/U6-AmCyan1. After *Bsa*I digestion of pENTR/U6-AmCyan1, non-targeting (NT) short hairpin RNAs (shRNAs) (shNT), *NEAT1*-targeting shRNAs (shNEAT1a/b), or *GABARAP*-targeting shRNAs (shGBRPa/b) were ligated with Ligation High ver.2 (Toyobo). The shRNA and AmCyan1-expressing cassettes were transferred by LR reaction to pAd/BLOCK-iT-DEST (Thermo Fisher Scientific). Adenovirus vectors were constructed by transfection of adenovirus plasmid DNAs into 293T cells with LipofectAMINE2000 (Thermo Fisher Scientific) according to the manufacturer’s protocol. The adenovirus titer was determined using the infectious genome titration protocol [55].

### 4.3. Reverse-Transcription Quantitative PCR (RT-qPCR) and Western Blot Analyses

RT-qPCR and Western blot analyses were performed as reported previously [10]. Protein and mRNA samples were prepared 48 h after seeding or irradiation. The primers used for RT-qPCR are found in Table S1. Relative mRNA expression levels were calculated using *β-actin* as the internal control. For Western blot analyses, antibodies recognizing GABARAP (sc-377300, Santa Cruz Biotechnology, Santa Cruz, CA, USA), glyceraldehyde-3-phosphate dehydrogenase (GAPDH; sc-365062, Santa Cruz Biotechnology), and β-tubulin (βTUB; sc-55529, Santa Cruz Biotechnology), as well as the Autophagy Antibody Sampler Kit (#4445, Cell Signaling Technology, Danvers, MA, USA), were used.

### 4.4. Colony Formation Assay

Cells were seeded into a 24-well plate and were allowed to attach overnight. The cells were irradiated (0, 1, 2.5, or 5 Gy) using an X-ray generator (MX-160Labo, mediXtec Japan, Chiba, Japan). Afterward, the medium was changed, or cells were transduced with adenovirus at a multiplicity of infection of 200. After 24 h in culture, 100–10,000 viable cells were quantified and seeded into a new plate. After 10 days, the cells were fixed and stained with a crystal violet solution (0.5% crystal violet in 10% methanol), and colonies (>50 cells) were counted.

### 4.5. Autophagic Flux Assay

Twenty-four hours after seeding or irradiation, cells were treated with 50 nM bafilomycin A1 (BafA; Cayman Chemical, Ann Arbor, MI, USA) for 24 h. We have confirmed that this treatment does not affect cell viabilities by a WST assay using Cell Counting Kit-8 ((Dojindo, Kumamoto, Japan) (data not shown). An equal volume of dimethyl sulfoxide (DMSO) was added to untreated control cells. LC3 puncta were assessed in HLF and HuH6 cells overexpressing mCherry-LC3. Cells were seeded onto a 3.5-cm glass-bottom dish (Matsunami Glass, Osaka, Japan) and were allowed to attach overnight. Cells were transduced with adenovirus vectors expressing shNT, shNEAT1a, or shNEAT1b. Confocal images of LC3-puncta (4 pictures for each shRNA) were obtained 48 h post-transduction using an LCV110CSU microscope (Olympus, Tokyo, Japan). The number of LC3-puncta was counted by ImageJ software (Bethesda, MD, USA).

### 4.6. Analysis of Gene Expression in HCC Tissues

The expression of *NEAT1v1* (ENST00000499732.2), *NEAT1v2* (ENST00000501122.2), and *GABARAP* (ENST00000302386.9) was analyzed in the Cancer Genome Atlas (TCGA) dataset for HCC (LIHC) by GEPIA2 [56].

### 4.7. Statistical Analysis

Three or more independent samples were analyzed in each experiment. All experimental values were expressed as means ± standard deviations. The differences between two groups were assessed by two-tailed unpaired Student’s *t*-test. Multiple comparisons were made using the two-tailed Dunnett’s or Tukey’s test. The differences were considered significant for a *p* value less than 0.05.

## Data Availability

The raw data are available upon request, please contact the corresponding author.

## Supplementary Materials

The following are available online at https://www.mdpi.com/article/10.3390/ijms23020711/s1.
