# Supplementary material for: NEAT1 Confers Radioresistance to Hepatocellular Carcinoma Cells by Inducing Autophagy through GABARAP
Source: Int J Mol Sci. 2022 Jan 10;23(2):711. doi: 10.3390/ijms23020711 (PMC8775719; doi:10.3390/ijms23020711)
Supplement: Supplementary file 1 [file ijms-23-00711-s001.zip › Table S1.pdf]

Table S1 Oligo DNAs used in the present study

**Oligo DNAs for adenovirus vector construction**	
BsaI-linker	
BsaI-U	CACCGGAGACCACGAATTCACACTGGGTCTCGTTTCCAGTAGATCTCCAG
BsaI-D	AAAACTGGAGATCTACTGGAAACGAGACCCAGTGTGAATTCGTGGTCTCC
AmCyan cloning primers	
AmCyan_For	GGACAGGGATCCGGTAAGCGGCAGGGT
AmCyan_Rev	GACGTTGGATCCACGTTCTTTAATAGTGGA
shNT	
shNT-U	CACCGGGCGCGATAGCGCTAATAATTTCTCGAGAAATTATTAGCGCTATCGCGCTTT
shNT-D	GAAAAAAGCGCGATAGCGCTAATAATTTCTCGAGAAATTATTAGCGCTATCGCGCCC
shNEAT1a	
shNEAT1a-U	CACCGGGGAGTCGGTATTGTTGGTAATCTCGAGATTACCAACAATACCGACTCCTTT
shNEAT1a-D	GAAAAAAGGAGTCGGTATTGTTGGTAATCTCGAGATTACCAACAATACCGACTCCCC
shNEAT1b	
shNEAT1b-U	CACCGGATGGACCGTGGTTTGTTACTACTCGAGTAGTAACAAACCACGGTCCATTTT
shNEAT1b-D	GAAAAAAATGGACCGTGGTTTGTTACTACTCGAGTAGTAACAAACCACGGTCCATCC
shGBRPa	
shGBRPa_U	CACCGGCACCATGAAGAAGACTTCTTTCTCGAGAAAGAAGTCTTCTTCATGGTGTTT
shGBRPa_D	GAAAAAACACCATGAAGAAGACTTCTTTCTCGAGAAAGAAGTCTTCTTCATGGTGCC
shGBRPb	
shGBRPb_U	CACCGGGTGCCTTCTGATCTCACAGTTCTCGAGAACTGTGAGATCAGAAGGCACTTT
shGBRPb_D	GAAAAAAGTGCCTTCTGATCTCACAGTTCTCGAGAACTGTGAGATCAGAAGGCACCC

**qPCR primers**		
Adenovirus titration	*forward*	*reverse*
AdE2B_Titer	TGTCAAGCTTGGTGGCAAAC	TCGCGACAAAAACCAAACCC
mRNA determination	*sense*	*antisense*
β-actin	GATGCAGAAGGAGATCACTGC	TGATCCACATCTGCTGGAAG
CD13	CTGCAGCAAAGAGTTGTGGATC	TTGCCCAATGACGTTGTTGG
CD90	TGAGATCCCAGAACCATGAACC	TATTCTCATGGCGGCAGTCC
CD44	AAGGTGGAGCAAACACAACC	TCGACTGTTGACTGCAATGC
CD133	AATTCACCAGCAACGAGTCC	AATCCATTCCCTGTGCGTTG
EPCAM	ATAACCTGCTCTGAGCGAGTG	AACGCGTTGTGATCTCCTTC
GABARAP	ACAATGGGTCAGCTGTACCAG	AGACCGTAGACACTTTCGTCAC
total NEAT1	TGCCACAACGCAGATTGATG	ACAAGAAGGCAGGCAAACAG
NEAT1v2	AGGGTTCTGTTGCTAACACG	TGCCGATGAAGCAACAAAGC
