# Supplementary figures and images for: NEAT1 Confers Radioresistance to Hepatocellular Carcinoma Cells by Inducing Autophagy through GABARAP
Source: Int J Mol Sci. 2022 Jan 10;23(2):711. doi: 10.3390/ijms23020711 (PMC8775719; doi:10.3390/ijms23020711)

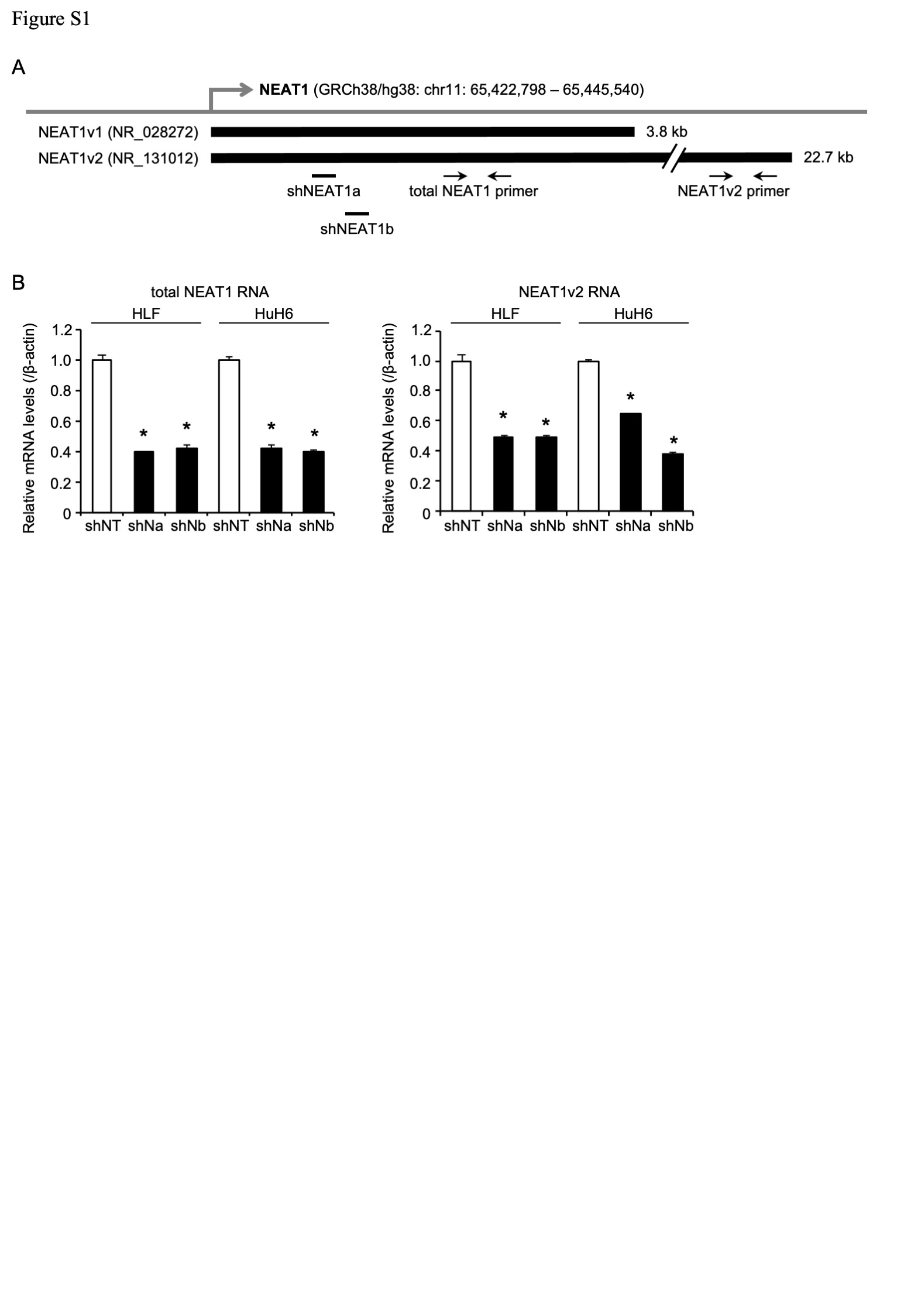

Supplement: Supplementary file 1 [file ijms-23-00711-s001.zip › Figure S1.jpeg]

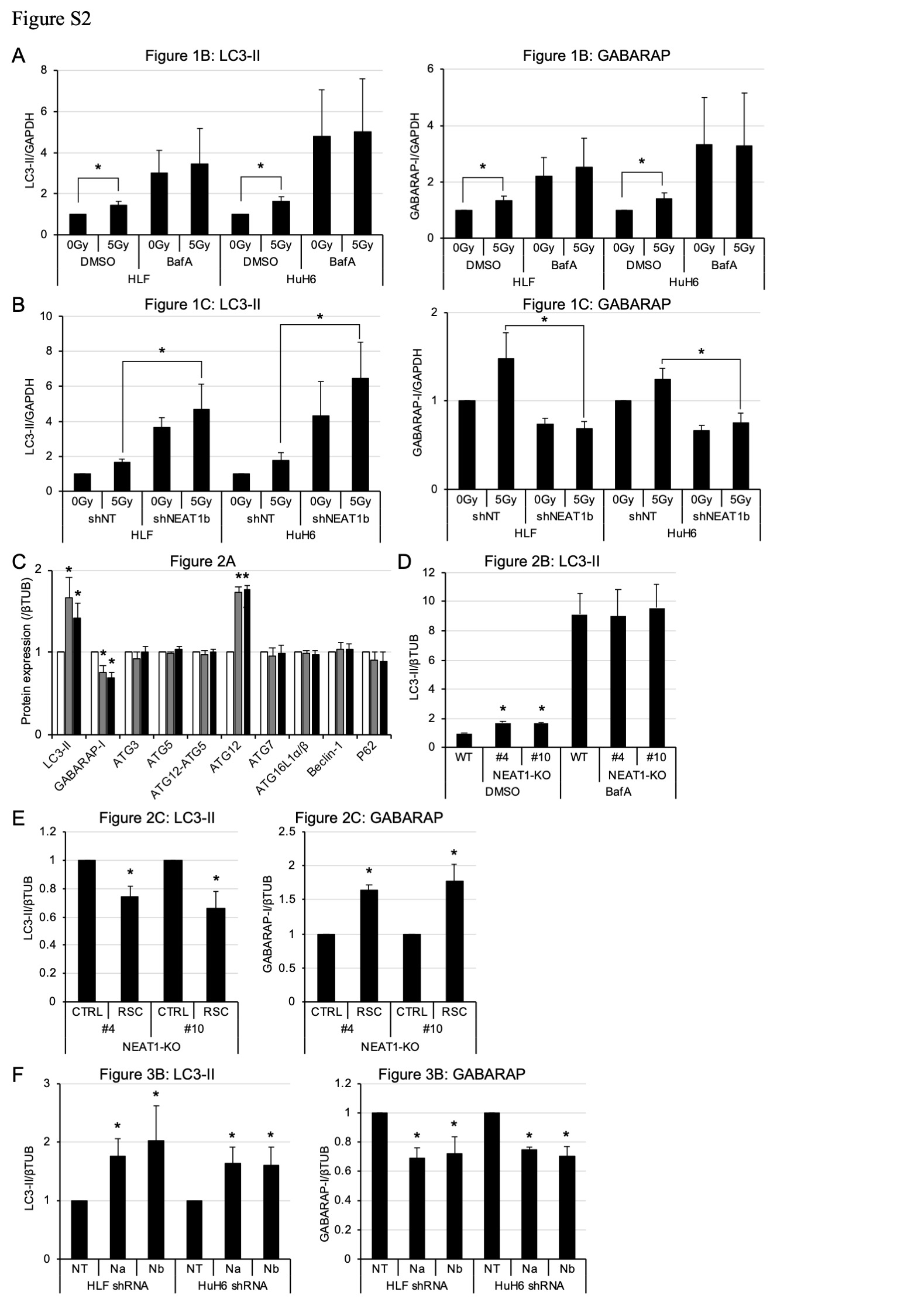

Supplement: Supplementary file 1 [file ijms-23-00711-s001.zip › Figure S2-1.jpeg]

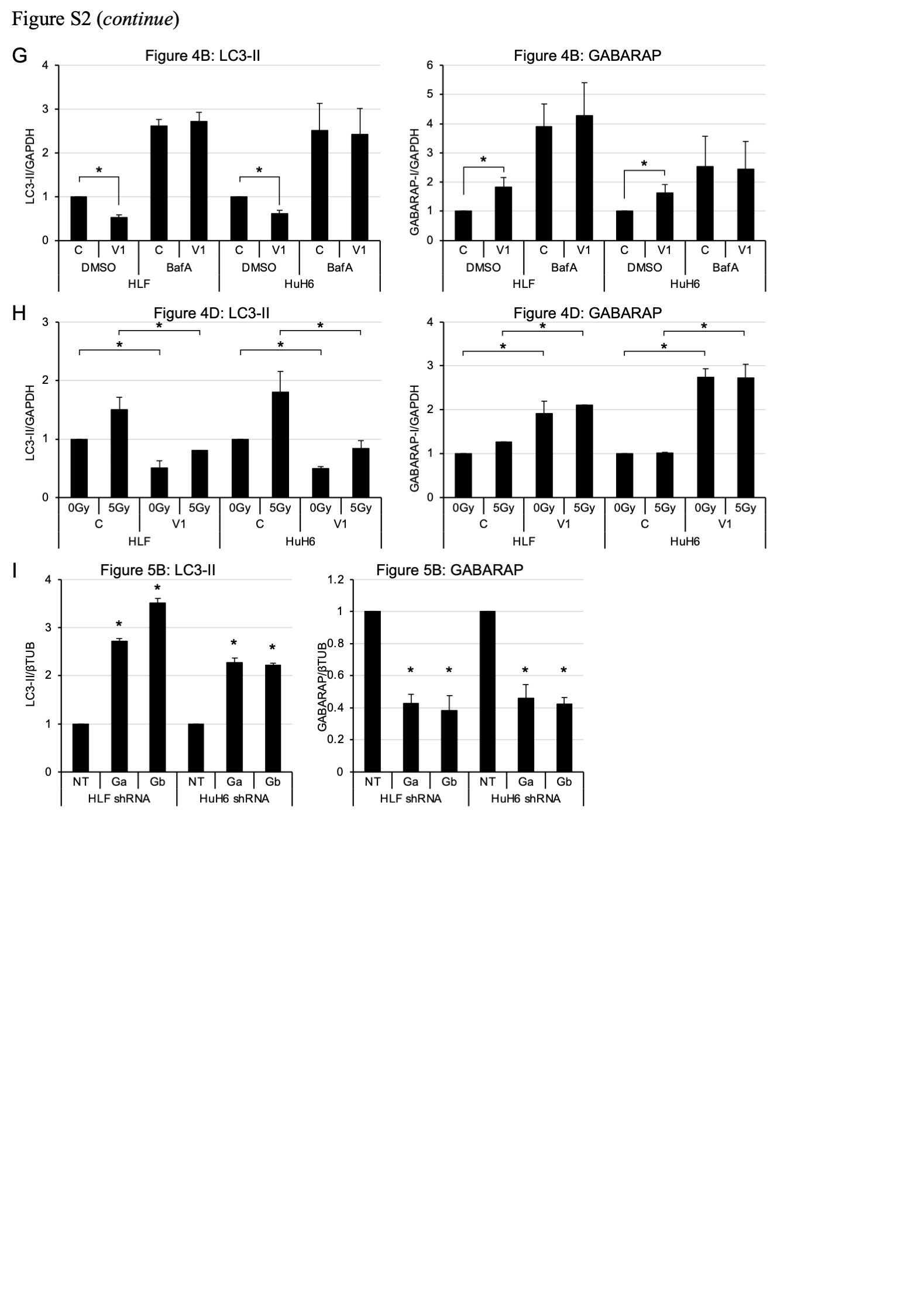

Supplement: Supplementary file 1 [file ijms-23-00711-s001.zip › Figure S2-2.jpeg]

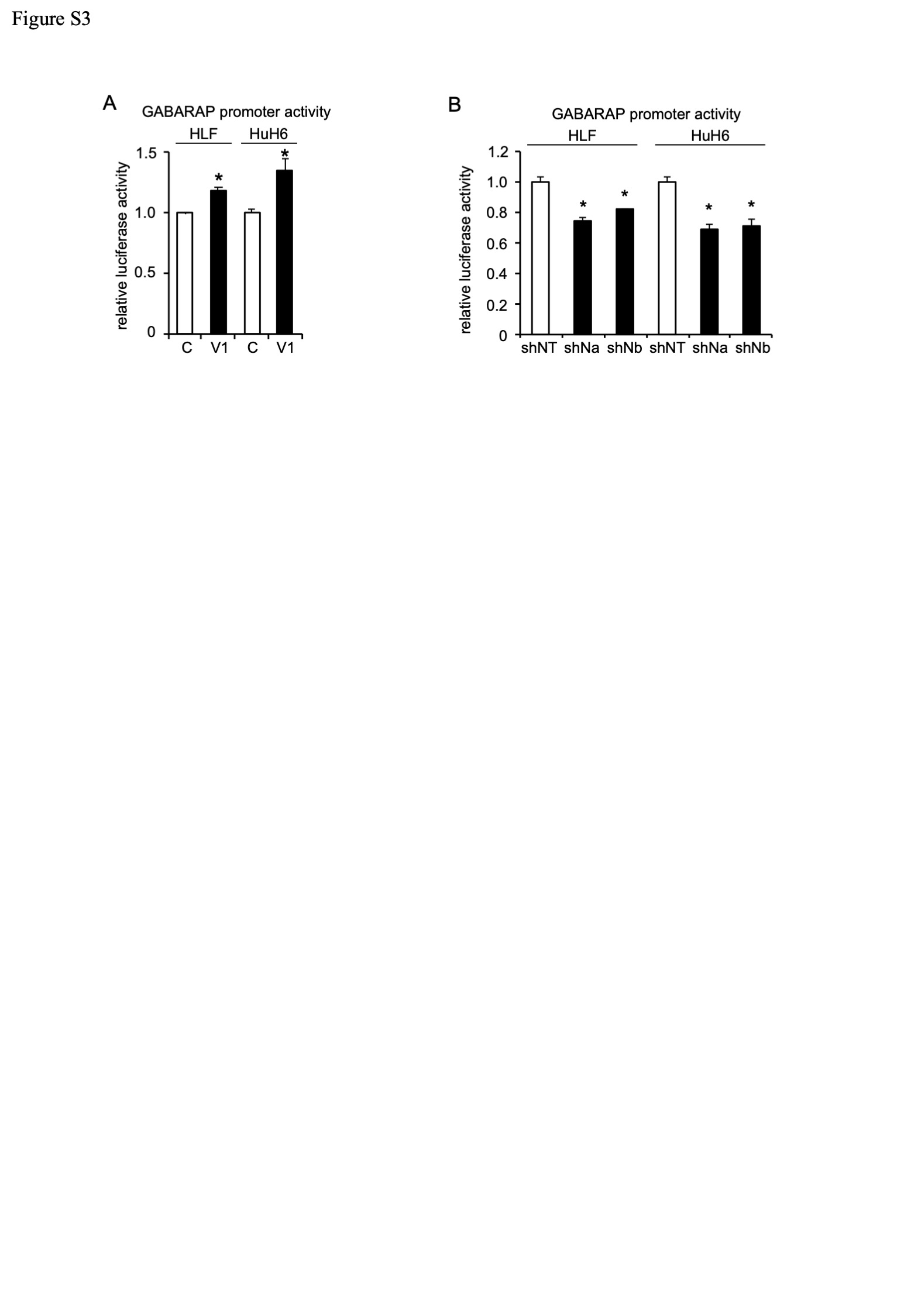

Supplement: Supplementary file 1 [file ijms-23-00711-s001.zip › Figure S3.jpeg]

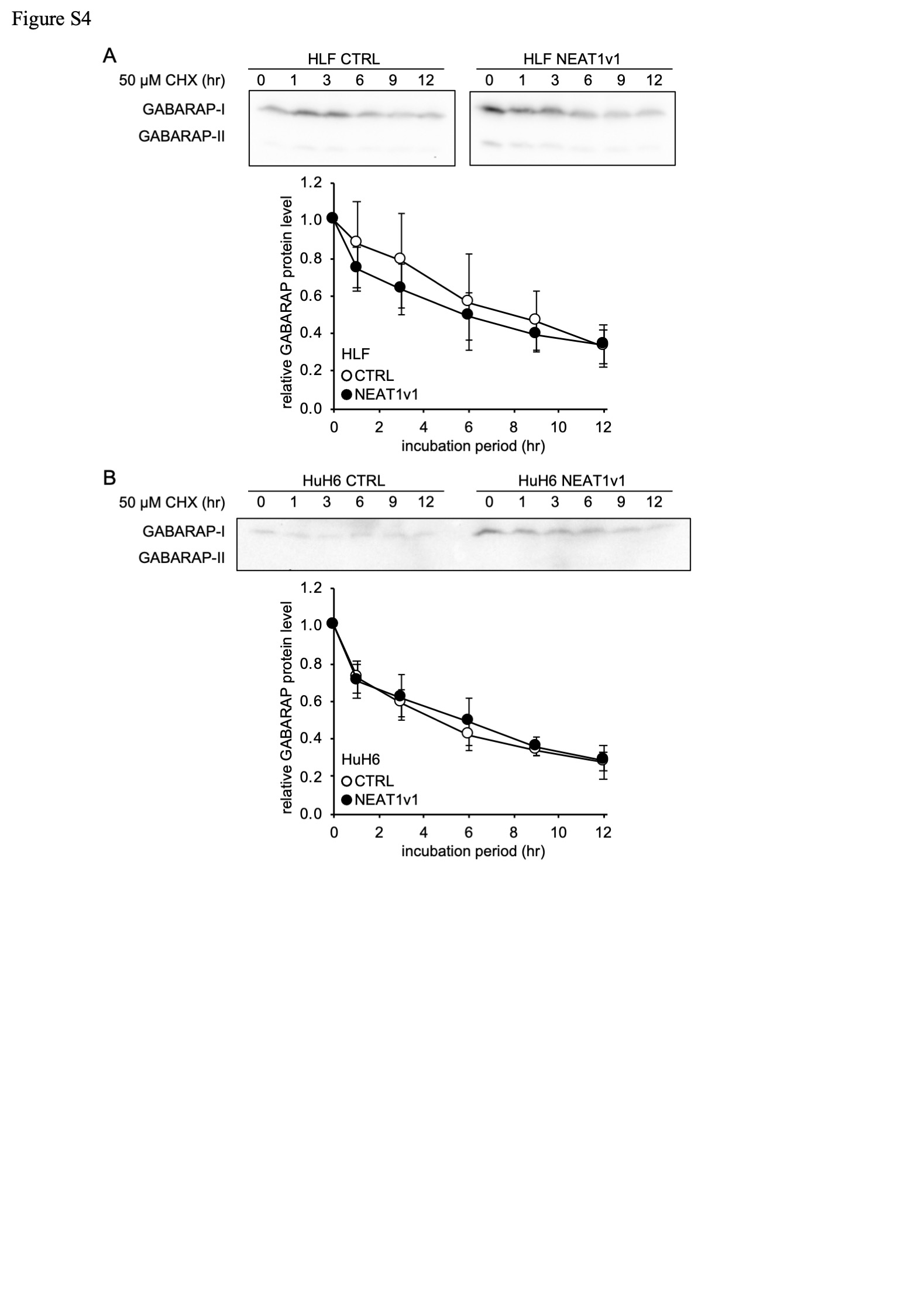

Supplement: Supplementary file 1 [file ijms-23-00711-s001.zip › Figure S4.jpeg]

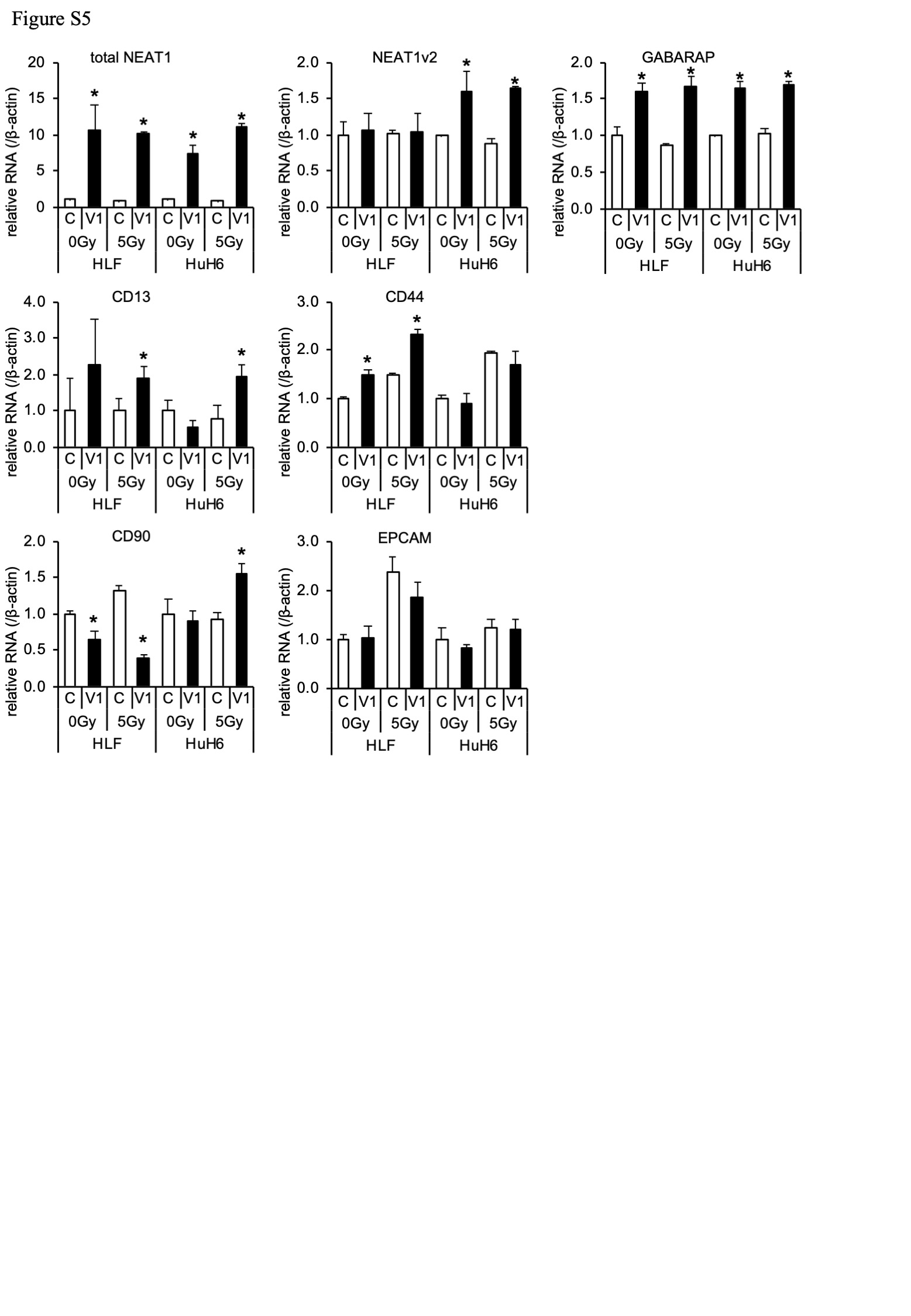

Supplement: Supplementary file 1 [file ijms-23-00711-s001.zip › Figure S5.jpeg]
